# Advancing environmental public health in Latin America and the Caribbean

**DOI:** 10.26633/RPSP.2021.118

**Published:** 2021-09-16

**Authors:** Marcelo Korc, Fred Hauchman

**Affiliations:** 1 Pan American Health Organization Washington, D.C. United States of America Pan American Health Organization, Washington, D.C., United States of America; 2 PAHO Climate Change and Environmental Determinants of Health Technical Advisory Group Virginia United States of America PAHO Climate Change and Environmental Determinants of Health Technical Advisory Group, Virginia, United States of America

**Keywords:** Climate change, environmental health, environment and public health, health equity, health policy, sustainable development, Americas, Cambio climático, salud ambiental, medio ambiente y salud pública, equidad en salud, política de salud, desarrollo sostenible, Américas, Mudança climática, saúde ambiental, meio ambiente e saúde pública, equidade em saúde, política de saúde, desenvolvimento sustentável, América

## Abstract

This paper highlights the important leadership role of the public health sector, working with other governmental sectors and nongovernmental entities, to advance environmental public health in Latin America and the Caribbean toward the achievement of 2030 Sustainable Development Goal 3: Health and Well-Being. The most pressing current and future environmental public health threats are discussed, followed by a brief review of major historical and current international and regional efforts to address these concerns. The paper concludes with a discussion of three major components of a regional environmental public health agenda that responsible parties can undertake to make significant progress toward ensuring the health and well-being of all people throughout Latin America and the Caribbean.

## RESUMEN

En este artículo se destaca el importante papel de liderazgo que tiene el sector de la salud pública, en colaboración con otros sectores gubernamentales y entidades no gubernamentales, en el fomento de la salud pública medioambiental en América Latina y el Caribe con el fin de alcanzar para el 2030 el Objetivo de Desarrollo Sostenible número 3: salud y bienestar. Se abordan las amenazas de salud pública medioambiental actuales y futuras más urgentes, y se presenta una breve revisión de las principales medidas históricas y actuales adoptadas a nivel regional e internacional para abordar estas cuestiones. El artículo finaliza con un análisis de los tres principales componentes de un programa regional de salud pública ambiental que las partes responsables pueden ejecutar para realizar progresos significativos en la garantía de la salud y el bienestar de todos en América Latina y el Caribe.

## RESUMO

Este artigo destaca o importante papel de liderança do setor de saúde pública, trabalhando com outros setores governamentais e entidades não governamentais, para promover a saúde pública ambiental na América Latina e no Caribe a fim de atingir o Objetivo de Desenvolvimento Sustentável 3 para 2030: Saúde e Bem-Estar. São discutidas as ameaças ambientais mais urgentes para a saúde pública, atuais e futuras, seguidas por uma breve revisão dos principais esforços históricos e atuais, internacionais e regionais, para abordar essas preocupações. O artigo conclui com uma discussão de três componentes essenciais de uma agenda regional de saúde pública ambiental que as partes responsáveis podem adotar para avançar significativamente no sentido de garantir a saúde e o bem-estar de todas as pessoas na América Latina e no Caribe.

Enhancements in health services, environmental protection, economic development, and other factors in recent decades have led to improvements in the health of people across Latin America and the Caribbean (LAC) (1). However, the negative health impacts associated with environmental hazards such as unsafe air and water, poor sanitation, and exposure to toxic substances continue to be of concern in many communities, particularly those that are poor and marginalized (2). Moreover, global environmental challenges such as climate change are increasingly having deleterious effects on the health and well-being of populations (3).

Negative health outcomes associated with the exposure to environmental threats are often a consequence of a combination of environmental, social, cultural, economic, and political factors. These factors can contribute to inequities in health and make a reduction in adverse health outcomes more difficult to achieve (1, 4). Outbreaks of disease, such as the coronavirus disease 2019 (COVID-19) pandemic, can exacerbate the situation by overwhelming health systems and causing severe negative effects on human health and economies.

The United Nations 2030 Agenda for Sustainable Development provides a blueprint to achieve a better and more sustainable future for all (5). The 17 goals of the Agenda are interconnected, and in order to leave no one behind, it is important that all are achieved. Sustainable Development Goal (SDG) 3 specifically addresses Health and Well-Being. In the context of environmental determinants of health, to achieve SDG 3, it is necessary to implement actions to achieve SDG 6 (Clean Water and Sanitation), SDG 7 (Affordable and Clean Energy), SDG 11 (Sustainable Cities and Communities), SDG 12 (Responsible Consumption and Production), and SDG 13 (Climate Action), among others.

This paper highlights the important leadership role of the public health sector, working with other governmental sectors and nongovernmental entities, to advance environmental public health in LAC toward the achievement of SDG 3.

## ENVIRONMENTAL PUBLIC HEALTH CHALLENGES

According to the World Health Organization (WHO), about 13% of premature deaths in high-income countries^1^ and 19% in low- and middle-income countries^2^ of the Americas are attributable to known, avoidable environmental risks, amounting to about 1 016 000 deaths each year (6, 7). With the adoption of the 2030 SDGs, United Nations Member States agreed that the most pressing environmental public health threats globally are air pollution, contaminated water, inadequate sanitation, poor management of solid waste, risks related to certain hazardous chemicals, and the negative impacts of climate change. Table 1 shows the most recent WHO estimates of mortality rates attributed to exposure to air pollution; unsafe water, sanitation, and hygiene services; and unintentional poisoning in the countries of the Americas.

### Air pollution

**FIGURE 1.**
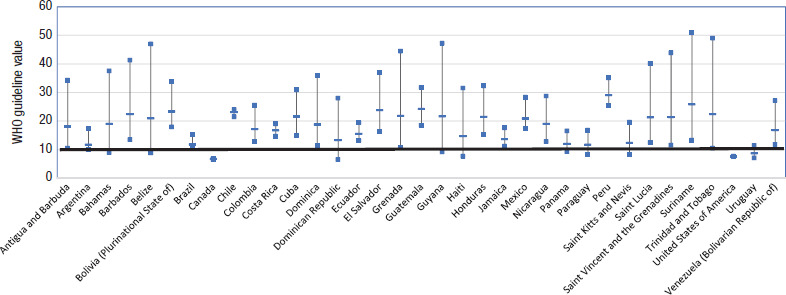
Ambient urban PM_2.5_ concentrations in the countries of the Americas in 2016 (annual average and 95% confidence interval)

***Source:*** Prepared by the authors using publicly available data from the WHO Global Health Observatory (https://www.who.int/data/gho/data/indicators/indicator-details/GHO/concentrations-of-fine-particulate-matter-(pm2-5)).

**FIGURE 2.**
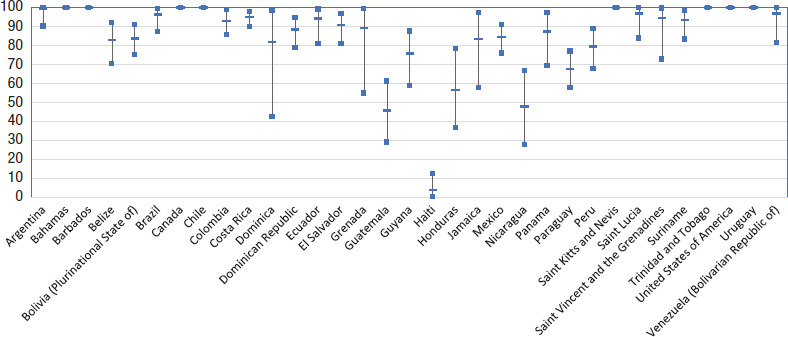
Percentage of population with primary reliance on clean fuels and technologies for cooking in the countries of the Americas in 2018 (annual average and 95% confidence interval)

***Source:*** Prepared by the authors using publicly available data from the WHO Global Health Observatory (https://www.who.int/data/gho/data/themes/air-pollution/household-air-pollution).

According to data for LAC collected by WHO (8), ambient and household air pollution was linked to approximately 250 000 premature deaths in 2016 due to stroke, heart disease, lung disease, and cancer. Riojas-Rodríguez et al. (9) found that only five of 104 cities in LAC that monitored for particulate matter with a diameter of 10 microns or less (PM_10_) and four of 57 that monitored for particulate matter with a diameter of 2.5 microns or less (PM_2.5_) complied with WHO air quality guidelines, resulting in the exposure of millions of people to unhealthy levels of pollution. In addition, almost 80 million people still depend on polluting fuels such as solid fuels or kerosene for lighting, cooking, and heating (10, 11). Figure 1 shows the ambient urban 2016 average PM_2.5_ concentrations and Figure 2 shows the proportion of population in 2018 with primary reliance on clean fuels and technologies for cooking in the countries of the Americas.

**TABLE 1.** WHO estimated mortality rates attributed to exposure to ambient and household air pollution; unsafe water, sanitation, and hygiene services; and unintentional poisoning in the Americas (annual average and 95% confidence interval)

Country	Mortality rate attributed to exposure to ambient and household air pollution (per 100 000 population, age-standardized), 2016			Mortality rate attributed to exposure to unsafe water, sanitation, and hygiene services (per 100 000 population), 2016			Mortality rate attributed to unintentional poisoning (per 100 000 population), 2019		
	Both sexes	Male	Female	Both sexes	Male	Female	Both sexes	Male	Female
Antigua and Barbuda	30 (25, 36)	37 (31, 44)	24 (20, 29)	0.1	0.1	0.1	0.7 (0.5, 1.0)	1.4 (0.9, 1.9)	0.2 (0.1, 0.2)
Argentina	27 (20, 35)	36 (27, 47)	20 (14, 27)	0.4	0.3	0.4	0.4 (0.3, 0.6)	0.5 (0.4, 0.8)	0.3 (0.2, 0.4)
Bahamas	20 (17, 24)	26 (22, 30)	15 (12, 19)	0.1	0.1	0.1	0.2 (0.1, 0.2)	0.2 (0.2, 0.4)	0.1 (0.1, 0.1)
Barbados	31 (25, 37)	39 (32, 46)	25 (20, 31)	0.2	0.1	0.2	0.7 (0.5, 1.0)	0.8 (0.5, 1.2)	0.6 (0.4, 0.9)
Belize	69 (57, 81)	83 (68, 98)	55 (45, 65)	1.0	1.0	0.9	0.4 (0.2, 0.5)	0.5 (0.3, 0.7)	0.2 (0.2, 0.3)
Bolivia (Plurinational State of)	64 (53, 76)	72 (60, 86)	56 (46, 66)	5.6	6.2	4.9	0.6 (0.2, 1.1)	0.6 (0.3, 1.3)	0.5 (0.2, 1.0)
Brazil	30 (24, 39)	37 (30, 49)	24 (19, 32)	1.0	1.0	1.1	0.1 (0.1, 0.2)	0.2 (0.2, 0.2)	0.1 (0.1, 0.1)
Canada	7 (5, 10)	9 (6, 12)	5 (3, 8)	0.4	0.3	0.4	0.3 (0.2, 0.4)	0.4 (0.3, 0.5)	0.3 (0.2, 0.3)
Chile	25 (19, 34)	33 (25, 44)	19 (14, 27)	0.2	0.2	0.2	0.4 (0.2, 0.5)	0.5 (0.3, 0.7)	0.2 (0.2, 0.3)
Colombia	37 (29, 47)	45 (36, 57)	30 (23, 38)	0.8	0.7	0.8	0.1 (0.1, 0.2)	0.2 (0.1, 0.3)	0.1 (0.0, 0.1)
Costa Rica	23 (18, 29)	29 (23, 36)	18 (14, 24)	0.9	0.7	1.0	0.1 (0.1, 0.1)	0.1 (0.1, 0.2)	0.1 (0.1, 0.1)
Cuba	50 (27, 96)	58 (33, 112)	42 (22, 83)	1.0	1.0	1.0	0.2 (0.1, 0.2)	0.2 (0.1, 0.3)	0.1 (0.1, 0.1)
Dominican Republic	43 (34, 53)	50 (40, 61)	36 (28, 45)	2.2	2.2	2.2	0.4 (0.2, 0.9)	0.5 (0.2, 1.1)	0.3 (0.1, 0.7)
Ecuador	25 (18, 34)	29 (21, 40)	21 (15, 29)	0.6	0.6	0.7	0.3 (0.2, 0.5)	0.4 (0.3, 0.7)	0.2 (0.1, 0.4)
El Salvador	42 (32, 52)	51 (40, 63)	35 (27, 44)	2.0	2.4	1.6	0.2 (0.1, 0.4)	0.4 (0.2, 0.7)	0.1 (0.1, 0.2)
Grenada	45 (37, 58)	52 (43, 63)	39 (31, 51)	0.3	0.7	<0.1	0.1 (0.1, 0.1)	0.0 (0.0, 0.0)	0.1 (0.1, 0.2)
Guatemala	74 (65, 83)	81 (70, 92)	68 (60, 76)	6.3	6.6	6.0	1.6 (1.0, 2.3)	2.3 (1.5, 3.3)	0.9 (0.6, 1.3)
Guyana	108 (88, 128)	117 (97, 140)	98 (80, 118)	3.6	4.1	3.2	0.1 (0.0, 0.1)	0.1 (0.0, 0.1)	0.1 (0.0, 0.1)
Haiti	184 (170, 198)	198 (180, 214)	172 (159, 185)	23.8	27.5	20.1	1.4 (0.4, 4.1)	1.9 (0.6, 6.5)	0.8 (0.2, 1.8)
Honduras	61 (51, 70)	76 (64, 87)	48 (40, 55)	3.6	3.4	3.8	0.5 (0.2, 1.0)	0.8 (0.3, 1.7)	0.1 (0.0, 0.2)
Jamaica	25 (19, 34)	30 (22, 40)	21 (15, 29)	0.6	0.7	0.6	0.1 (0.0, 0.1)	0.1 (0.1, 0.1)	0.1 (0.0, 0.1)
Mexico	37 (31, 43)	44 (37, 52)	30 (25, 36)	1.1	1.0	1.1	0.4 (0.3, 0.5)	0.6 (0.4, 0.7)	0.3 (0.2, 0.3)
Nicaragua	56 (48, 63)	63 (54, 72)	50 (43, 57)	1.9	2.1	1.7	0.3 (0.1, 0.5)	0.4 (0.2, 0.7)	0.2 (0.1, 0.3)
Panama	26 (20, 33)	31 (24, 40)	21 (15, 27)	2.2	2.4	2.0	0.1 (0.1, 0.1)	0.1 (0.1, 0.2)	0.1 (0.0, 0.1)
Paraguay	57 (47, 67)	66 (54, 79)	49 (40, 58)	1.5	1.4	1.5	0.2 (0.1, 0.4)	0.3 (0.1, 0.5)	0.1 (0.1, 0.3)
Peru	64 (52, 75)	74 (61, 88)	55 (45, 65)	1.3	1.3	1.2	0.4 (0.2, 0.7)	0.5 (0.2, 0.9)	0.3 (0.1, 0.5)
Saint Lucia	30 (25, 36)	36 (30, 43)	25 (20, 31)	0.6	1.0	0.3	0.1 (0.1, 0.2)	0.2 (0.1, 0.3)	0.1 (0.1, 0.1)
Saint Vincent and the Grenadines	48 (38, 60)	56 (46, 69)	40 (31, 51)	1.3	1.4	1.3	0.0 (0.0, 0.0)	0.0 (0.0, 0.0)	0.0 (0.0, 0.0)
Suriname	57 (47, 68)	74 (62, 88)	42 (35, 51)	2.0	2.1	2.0	0.3 (0.1, 0.6)	0.3 (0.1, 0.8)	0.3 (0.1, 0.5)
Trinidad and Tobago	39 (33, 45)	49 (42, 56)	30 (25, 36)	0.1	0.2	0.1	0.1 (0.1, 0.2)	0.1 (0.1, 0.2)	0.1 (0.0, 0.1)
United States of America	13 (10, 18)	17 (12, 21)	10 (7, 15)	0.2	0.2	0.3	0.5 (0.4, 0.6)	0.7 (0.6, 0.7)	0.3 (0.3, 0.4)
Uruguay	18 (13, 24)	25 (19, 34)	12 (9, 18)	0.4	0.3	0.4	0.5 (0.3, 0.7)	0.6 (0.4, 0.8)	0.4 (0.3, 0.5)
Venezuela (Bolivarian Republic of)	35 (28, 43)	45 (37, 55)	26 (21, 33)	1.4	1.4	1.4	0.2 (0.1, 0.4)	0.3 (0.2, 0.5)	0.2 (0.1, 0.3)

***Source:*** Prepared by the authors using publicly available data from the WHO Global Health Observatory (https://apps.who.int/gho/data/view.sdg.3-9-data-ctry?lang=en).

### Contaminated water and inadequate sanitation

**FIGURE 3.**
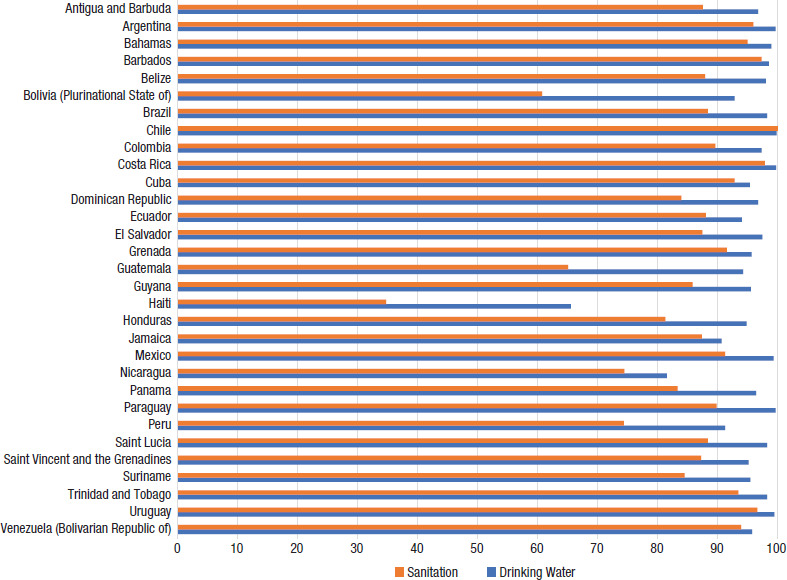
Household percent coverage of at least basic drinking water and sanitation services in LAC countries in 2017

***Note:*** Argentina data are only for urban areas.

***Source:*** Prepared by the authors using publicly available data from the WHO UNICEF Joint Monitoring Program (https://washdata.org/data/household#!/).

The human health impacts of poor drinking-water quality and inadequate sanitation are well documented. In their review of pollution and disease, the *Lancet* Commission (2) reported that acute and chronic gastrointestinal illness, particularly diarrhea, is the most commonly observed effect, followed by typhoid fever, paratyphoid fever, and lower respiratory tract infections. Exposure of susceptible individuals to hazardous chemicals in water is associated with a variety of short- and long-term adverse effects on health. In LAC, over 19 million people (~3% of the total population) did not have at least basic drinking water services in 2017. Nearly 84 million people (about 13% of the total population) had inadequate sanitation services, of which 13 million practiced open defecation (12). A greater proportion of people with inadequate services live in rural areas, particularly in less developed parts of the region (13). Figure 3 shows the household coverage of at least basic drinking water and sanitation services in LAC countries in 2017. Degradation of local water quality in coastal and inland areas of the region due to the rapid growth of toxic algae has caused serious harm to people, animals, and the aquatic environment, as well as negative impacts on the local economy. This problem is likely to become more widespread with increasing climate variability and uncontrolled pollution of waterbodies (14).

### Inadequate solid waste management

A lack of basic solid waste collection services and open dumping of waste exists in parts of LAC, along with indiscriminate dumping of waste in streams or abandoned areas. The inadequate management of solid waste can lead to contamination of the environment and serious health threats to communities and those who work at waste sites (15).

### Exposure to hazardous chemicals

WHO estimates that acute poisonings and exposure to toxic chemicals with primarily longer term effects are responsible for 1.6 million deaths and 45 million disability adjusted life-years lost globally (16). In LAC, exposure to toxic chemicals such as lead, mercury, arsenic, and pesticides in the air, water, soil, and food is an important contributor to disease and is of particular concern to children and other highly susceptible populations (17, 18). Previously unknown or poorly characterized contaminants in environmental media, such as novel insecticides and nanomaterials, have raised new concerns (2).

### Negative climate change-related impacts

This urgent global environmental concern is increasingly having adverse impacts on the health and well-being of populations in LAC and across the globe (19). The morbidity and mortality associated with the effects of a changing climate are likely to be exacerbated in areas where poverty, population pressures, and inadequate public health infrastructure exist. Other impacts include food insecurity, changes in patterns of infectious disease transmission, and threats to mental health. The issue of climate change is of particular concern to the small island states, for which extreme weather events can be particularly devastating (20).

A critical challenge the LAC region faces is health inequalities between and within countries. To analyze and develop recommendations to address this issue, the Pan American Health Organization (PAHO) Commission on Equity and Health Inequalities in the Americas (1) developed a framework with an explicit focus on health as a fundamental human right. The framework identifies political, social, cultural, economic, and environmental factors including climate change, as well as colonialism and racism toward Indigenous populations and people of African descent, as structural factors that contribute to health inequities in the Americas. It describes conditions of daily life (e.g., life stage, work and income, housing conditions) that impact health equity, and highlights the influence that gender, ethnicity, and other bases of discrimination may have on health outcome. The Commission developed 12 recommendations, each with multiple objectives and specific actions to be taken by governments, civil society, communities, and international organizations to achieve health equity.

## EVOLUTION OF ENVIRONMENTAL PUBLIC HEALTH IN LAC

Since the beginning of the 20th century, the environmental conditions in which people live, work, and play have been recognized as important contributors to the physical, mental, and emotional health of people in communities across LAC. Unsafe drinking water and inadequate sanitation services were among the earliest environmental public health concerns, whereas the potential for major impacts on health and security due to climate change-related impacts and degradation of ecosystems emerged as major concerns in the 21st century. In response to these concerns, efforts have been initiated at the international, regional, and national levels to protect the health and safety of people from environmental threats.

During the first four decades of the 20th century, issues related to the expansion and improvement of sanitation to combat epidemics were an important focus of the deliberations of what is known today as PAHO (21). Sanitation was also one of the main public health priorities of WHO when it was established in 1948 to promote health and well-being globally.

In the 1950s, drinking water coverage in LAC reached 60% of the urban population and 8% of the rural population, while sanitation services reached only 28% in urban areas and were almost nonexistent in rural areas. With the support of the Alliance for Progress and Special Fund for Public Water Supply in the 1960s (22), drinking water coverage reached 78% of the population in urban areas, whereas sanitation services reached only 38%. In rural areas, however, advances were less significant (21). Throughout this period, environmental public health services were under the responsibility of national health entities.

The United Nations Environment Programme (UNEP) was created following the 1972 United Nations Conference on Human Environment in Stockholm, Sweden (23), and numerous national and international environmental movements have since emerged. These movements have been divided into three interrelated but separate efforts: conservation, environmentalism, and public health. Most of these efforts have been in the areas of conservation and environmentalism. With UNEP’s support, LAC countries started to create national environmental protection authorities and establish environmental protection laws (24).

Beginning in the 1970s, human resources and responsibilities for some environmental health functions were transferred from the national health sector to environmental protection and water and sanitation entities. The reduction in human and financial resources for environmental public health programs and institutions impacted the ability of the public health sector to anticipate, recognize, and respond to potential environmental health threats.

In the 1980s, the United Nations General Assembly launched the International Drinking Water Supply and Sanitation Decade (1981–1990) as an ambitious global initiative to improve access to water and sanitation services. The results achieved by this initiative in LAC were modest due to deteriorated economic conditions and political changes. Coverage of water services in urban and rural areas reached 88% and 55%, respectively, whereas coverage of sanitation services reached 80% and 32%, respectively (25). The outbreak of cholera in the region in early 1991 showed the urgent need to focus not only on coverage but also on the quality of water services (26).

Since the late 1980s, high-income country governments and international financial institutions have advocated for privatization of water and other public services in LAC to combat inadequate financing for infrastructure and inefficiency in the provision of services. There has been little consensus on the improvement of water services due to this initiative. From the public health perspective, it may have contributed to inequities in health and hampered the development of public health capacities in the region (27).

In 1992, governments from around the world adopted an action plan for sustainable development, called Agenda 21, at the United Nations Conference on Environment and Development (the Earth Summit) in Rio de Janeiro, Brazil. Agenda 21 provided a global context for actions to improve environmental public health and promoted sustainability as a central concept for addressing public health concerns associated with exposure to environmental threats (28, 29).

A key initiative toward sustainable development was the United Nations Millennium Declaration in 2000 (30), and the adoption of the eight Millennium Development Goals (MDGs), with measurable targets to be achieved by 2015. Priority was given to the obligations contracted in the Stockholm, Rotterdam, and Basel Conventions for the safe handling of hazardous chemical substances. While most of the MDG targets were met, gaps and challenges remained (31), manifestations of the continued inequalities in the region.

In 2015, all United Nations Member States adopted the SDGs, as part of the 2030 Agenda for Sustainable Development (5). Unlike the MDGs, only one of the 17 SDGs specifically addresses health, five address environmental determinants of health, and most of the others address social determinants. In the Americas, at the request of its Member States, PAHO developed a strategic approach to achieve the SDGs, seeking collaboration among programs and partners, avoiding duplication, focusing on country needs, and placing health at the center as an indispensable contributor to a sustainable and equitable world (32). Efficient and effective environmental public health systems and delivery of services to all people may be one of the greatest challenges facing the national governments of the region to address the environmental determinants of health SDGs (33).

## ACTIONS TO ADVANCE ENVIRONMENTAL PUBLIC HEALTH IN LAC

To assist countries in the region to address the stated environmental public health challenges, the Director of PAHO created the Climate Change and Environmental Determinants of Health Unit in 2018. A Technical Advisory Group (TAG) was established to guide the technical cooperation of the Unit toward achieving SDG 3, Good Health and Well-Being.

In 2019, the World Health Assembly approved the WHO Global Strategy on Health, Environment and Climate Change (3). The strategy envisions an integrated and evidence-informed approach that fosters the leadership role of the health sector, with a focus on reducing inequities in health and promoting environmental sustainability.

After several TAG consultations and a consensus-driven decision-making process with Member States during 2018–2020, PAHO established a regional environmental public health agenda (34). Toward the achievement of the SDGs under the umbrella of the WHO global strategy, and building upon the commitments set forth in the Sustainable Health Agenda for the Americas 2018–2030 (35) and the Strategic Plan of PAHO 2020–2025 (36), the agenda focuses on the following three major areas (see Table 2).

### 1. Improving the performance of environmental public health programs and institutions

Strong and effective environmental public health programs and institutions are essential for achieving meaningful reductions in the burden of disease associated with environmental risks. The ability of a health system to fulfill this key role depends on good governance, effective implementation of key functions, and a workforce at national, state, and local levels with strong technical and interpersonal skills. The *Lancet* Commission on Pollution and Health (2) and *Lancet* Countdown on Climate Change and Health (37) offered several recommendations that have implications for the ability of public health programs to be effective.

**TABLE 2.** Priority areas identified in the Agenda for the Americas on Health, Environment, and Climate Change 2021–2030

Priority areas	Objectives
Improving the performance of environmental public health programs and institutions	Strengthen environmental public health surveillance, public policies, and knowledge management. Enhance health sector collaboration with other sectors to address environmental determinants of health. Strengthen the technical capacity of the health sector on environmental public health.
Fostering environmentally resilient and sustainable health systems	Provide resilient environmental infrastructure and services in health care facilities. Reduce the environmental footprint of health care facilities.
Promoting environmentally resilient and healthy communities	Integrate environmental public health in local health protection and improvement programs. Integrate environmental public health in local development programs outside the health sector. Strengthen environmental public health capacity for emergency and disaster response and early recovery.

***Source:*** Adapted by the authors from the PAHO Agenda for the Americas on Health, Environment, and Climate Change 2021–2030 (34)

According to WHO, good governance in health refers to political processes that reflect the values and principles that will contribute most effectively to economic and social development, including the progressive realization of the right to health. To exercise good governance in health, LAC countries should focus on strengthening the capacity of governments/decisionmakers to develop and implement strategies toward achieving SDG 3. In the context of environmental determinants of health, emphasis should be placed on air quality, chemical safety, climate change-related impacts, solid waste management, and water and sanitation factors that influence human health and their related behaviors. A context-specific health equity lens should be applied, using inter-programmatic approaches and intersectoral approaches.

Key services provided by a public health system are commonly referred to as essential public health functions (EPHFs). A growing appreciation for a more expanded view of EPHFs with a greater focus on equity in health has emerged in the public health community. For LAC countries, PAHO recently published a renewed conceptual framework for EPHFs (38). Assessments of EPHFs in environmental public health at national and subnational levels can be useful for determining the effectiveness of key environmental public health services in achieving their intended goals and identifying where additional actions and resource investments are warranted. The responsibility for the assessments must be shared among decisionmakers and partners; the assessments must be fully integrated into the standard policy cycle, with high-level support and resource commitments; and policymakers must receive expert technical assistance from domestic and/or international organizations.

A health workforce with strong technical and interpersonal skills is vital for the success of a health system’s programs and institutions. Staff should be adequate in number and trained and certified where appropriate. To achieve this objective in the context of environmental public health, countries may consider the use of standardized environmental public health professional qualifications, educational requirements, and credentialing using national and/or international professional organizations (39).

### 2. Fostering an environmentally resilient and sustainable health system

Health systems are both affected by and can contribute to environmental degradation. For example, health care facilities can be affected by an unstable and changing climate and other environmental stresses. Hurricanes and floods can affect infrastructure, and limited access to safely managed water and sanitation services can affect the quality of services. At the same time, health care facilities use resources and release waste into the environment. Increasing the resilience of a health system to climate change-related impacts and other environmental stresses, and minimizing the use of resources and generation of waste, may contribute to higher quality of care, accessibility of services, and reduction of costs (40).

There are several initiatives to promote environmentally sustainable and resilient health systems. For example, PAHO’s Smart Hospitals Toolkit (41) is a series of instruments designed to ensure health care facilities are resilient (specifically to disasters) and environmentally sustainable. Canada and the United States of America have been active in developing strategies to promote environmentally sustainable and resilient health systems, focusing specifically on health care facilities (42). Another relevant initiative is the Global Green and Healthy Hospitals project of Health Care Without Harm (43). The National Health Service in the United Kingdom has been leading this process and is the first in the world to commit to achieving net zero carbon emissions by 2040 (44).

WHO recently published a guidance report that focuses on the fundamental requirements to build climate resilience and support environmentally sustainable practices in health care facilities (40). It also calls for supporting a safe and informed health workforce. The guidance is particularly relevant to small and medium-size facilities and low to middle income countries.

### 3. Promoting environmentally resilient and healthy communities

Due to the many interrelated factors that need be addressed to enable communities to be environmentally healthy and resilient, WHO and others have promoted an intersectoral approach referred to as Health in All Policies, in which the health sector works with other sectors to create changes in policies to promote and protect health. This approach enables governments to better address social and environmental determinants of health and implement actions with the community for reducing inequities in health (45). In addition, there is a need for inter-programmatic coordination and integration within the health sector to control and prevent diseases (46).

The social, political, economic, and environmental challenges faced by Indigenous populations and people of African descent make it particularly important for these groups to be included in decisions and actions that impact their health. Examples of environmental advocacy and involvement by Indigenous groups in several LAC countries (2)^3^ with governmental and nongovernmental entities highlight the point that efforts to achieve health equity and ensure human rights can only be accomplished if these special communities are meaningfully engaged in both the assessments of environmental concerns and the identification of solutions.

Several challenges must be overcome in order for inter-programmatic and intersectoral approaches to be successful, not the least of which are resource constraints and competing priorities, lack of institutional capacity, weak governance, and political issues. The need for meaningful engagement of multiple sectors requires strong leadership and extensive coordination, communication, and the breakdown of institutional barriers. Furthermore, to be truly effective in reducing the unequal distribution of health benefits across a whole population, poverty and other structural causes of inequities in societies must be addressed (1).

Notwithstanding these challenges, well-managed, people-centered, context-specific and comprehensive integrated actions in which there is a shared vision, active engagement of all stakeholders, and good communication between coordinating entities can be effective in reducing the burden of disease attributed to environmental factors. Notable mechanisms supporting these actions, particularly at the local level, include the establishment of coordination structures (e.g., committees, councils), the use of planning and priority-setting processes, access to financial tools and effective approaches for identifying and prioritizing risks, and the existence of mandates for action and accountability (47). Monitoring and evaluation of processes and outcomes associated with these actions are important mechanisms for taking corrective measures and sharing lessons learned.

## CONCLUSIONS

This paper highlights the important leadership role of the public health sector, working with other governmental sectors and nongovernmental entities, to advance environmental public health in LAC toward the achievement of SDG 3. The health sector plays an important role in leading efforts to strengthen the performance of environmental public health programs and institutions. Good governance, effective implementation of key functions, and a skilled workforce are essential. Strong environmental public health programs and institutions strengthen the capacity of governments/decisionmakers to develop and implement strategies toward achieving SDG 3. In addition, an environmentally resilient and sustainable health system may contribute to higher quality of care, accessibility of services, and reduction of costs. Moreover, environmentally resilient and healthy communities may bring economic benefits and provide social and environmental benefits. This becomes even more urgent during health emergencies.

The commitment of governments and communities to fully adopt a human rights approach to health, as well as the leadership of the public health community, will determine the extent to which equity in health as it relates to environmental public health will be achieved for all people. Technical collaboration among countries, along with technical support from national, regional, and global organizations, can be effective means of strengthening institutional capacities for solving environmental public health concerns. Finally, emphasis should be placed on inter-programmatic and intersectoral approaches to mainstream interventions to address environmental determinants of health in processes and programs within and outside the health sector that are not focused solely on health.

## Disclaimer.

Authors hold sole responsibility for the views expressed in the manuscript, which may not necessarily reflect the opinion or policy of the *RPSP/PAJPH* and/or PAHO.

## Footnotes

1 High-income countries: Antigua and Barbuda, Bahamas, Barbados, Canada, Chile, Saint Kitts and Nevis, Trinidad and Tobago, United States of America, Uruguay.

2 Low- and middle-income countries: Argentina, Belize, Bolivia (Plurinational State of), Brazil, Colombia, Costa Rica, Cuba, Dominica, Dominican Republic, Ecuador, El Salvador, Grenada, Guatemala, Guyana, Haiti, Honduras, Jamaica, Mexico, Nicaragua, Panama, Paraguay, Peru, Saint Lucia, Saint Vincent and the Grenadines, Suriname, Venezuela (Bolivarian Republic of).

3 See pages 58, 65, 193, and 255, respectively, of the report of the PAHO Commission on Equity and Health Inequalities in the Americas (1).
